# Supplementary material for: Curated and harmonised transcriptomics datasets of interstitial lung diseases
Source: Data Brief. 2025 Oct 14;63:112139. doi: 10.1016/j.dib.2025.112139 (PMC12581653; doi:10.1016/j.dib.2025.112139)
Supplement: Supplementary file 1 [file mmc1.zip › Supplementary_material/RNA-seq/fastQC reports/GSE169500/SRR14057119_fastqc.html]

SRR14057119.fastq.gz FastQC Report 

FastQC Report

Mon 5 Feb 2024  
SRR14057119.fastq.gz

## Summary

- Basic Statistics
- Per base sequence quality
- Per sequence quality scores
- Per base sequence content
- Per sequence GC content
- Per base N content
- Sequence Length Distribution
- Sequence Duplication Levels
- Overrepresented sequences
- Adapter Content

## Basic Statistics

| Measure | Value |
| --- | --- |
| Filename | SRR14057119.fastq.gz |
| File type | Conventional base calls |
| Encoding | Sanger / Illumina 1.9 |
| Total Sequences | 12295986 |
| Sequences flagged as poor quality | 0 |
| Sequence length | 25-371 |
| %GC | 50 |

## Per base sequence quality

## Per sequence quality scores

## Per base sequence content

## Per sequence GC content

## Per base N content

## Sequence Length Distribution

## Sequence Duplication Levels

## Overrepresented sequences

| Sequence | Count | Percentage | Possible Source |
| --- | --- | --- | --- |
| ACCAATGTCATAGGGTGCAATATCTACAATAGGTAGTCTCACAGCCTTGC | 131695 | 1.0710405818614301 | No Hit |
| ACACTGAGGACTCTGTTCCTCCCCTTTCCGCCTAGGGGAAAGTCCCCGGA | 127720 | 1.0387129588468953 | No Hit |
| GGGAATGTCTACGTGCGTATGCACGTGGCACTCTCTGCCCGAGGTCCGGG | 126640 | 1.0299296046693611 | No Hit |
| AGAGCATGACCGATGGATTCCAGTTCGAGTATGGCGGCCAGGGCTCCGAC | 119463 | 0.9715609630655077 | No Hit |
| CAATATCAAGGAAGGGCAGGCGTGATGGCTTATTTGTTTTGTATTCAATG | 113907 | 0.926375485463305 | No Hit |
| GGGCCACGAGCTGAGTGCGTCCTGTCACTCCACTCCCATGTCCCTTGGGA | 92179 | 0.7496674117878793 | No Hit |
| ATGTTCTGGGAGGCCTCGGTGGACATCAGGCGCAGGAAGGTCAGCTGGAT | 85581 | 0.6960076239514261 | No Hit |
| TTCTTGTAGATGGCTGCTCTAAAAAGACAAATGAATGGGGAAAGACAATC | 74585 | 0.6065800660475703 | No Hit |
| TGGTTGCACGAAACACACTGGGGAATGGAGCAAAACAGTCTTTGAATATC | 68493 | 0.5570354422979987 | No Hit |
| AAGCTCAGGGAGAGCCCTGTTAGGGCCGCCTCTGGCCCTAGTCTCAGACC | 53915 | 0.43847642637198836 | No Hit |
| TGAAGCTCAGGGAGAGCCCTGTTAGGGCCGCCTCTGGCCCTAGTCTCAGA | 38716 | 0.314866981793896 | No Hit |
| TGGTTCCTGGCAAGCCCATGTGTGTTGAGAGCTTCTCAGACTATCCACCT | 37530 | 0.3052215576693077 | No Hit |
| ACACCCACCGCAACTGTCTGTCTCATATCACGAACAGCAAAGCGACCCAA | 33474 | 0.2722351830914576 | No Hit |
| GCTGCTACAGGAGAATAGCAGACAGGTATAGTTAAGAAACAACTTTATTT | 28427 | 0.23118926778218518 | No Hit |
| AAGAGAAAGCAGTGCAAACCTTCCCGTGGCCGCAAGCGTGGCATCTGCTG | 27670 | 0.2250327871225618 | No Hit |
| AGCGGTTCCGCTGCCCTGAGGCACTCTTCCAGCCTTCCTTCCTGGGCATG | 27587 | 0.2243577700885476 | No Hit |
| TCACACTTCATGATGGAGTTGAAGGTAGTTTCGTGGATGCCACAGGACTC | 26553 | 0.21594852173709372 | No Hit |
| CAGAAGCTCTTTATGTCAAACTTCTCAAGTTGATTGACCTCCAGGTAATA | 26125 | 0.21246771100747835 | No Hit |
| TCAACGTACTCCATGCCTGGCAGCTTCATCCCGTACTTGTCCACGCACCA | 25811 | 0.20991403210771387 | No Hit |
| TGTTCCCTCTCCTCATGAGATTGGTGAAGAAAGTATTTGGCAAAGTTCTT | 22285 | 0.18123800726513514 | No Hit |
| ACCCGCAGCTTCTGCTTCTCAGTCAGAAGGTTGTTGTCCTCATCCCTCTC | 21861 | 0.17778972747691807 | No Hit |
| TGTGCCCTGGAAGCGGAATGGAAACTTGCAGGGCTGTCCTTCAGCGTTGC | 21068 | 0.17134046834471023 | No Hit |
| TCTGCAATGCAATCACAATGCCCAAACTAGACCTGCCATTTCTCACACAC | 21024 | 0.1709826279893292 | No Hit |
| ATGTTCTGGGAGCCTCGGTGGACATCAGGCGCAGGAAGGTCAGCTGGATG | 20955 | 0.17042146925020896 | No Hit |
| CAGTAGCACCAACGTTGATGAGGTCGTGAGTCAGGGGTCCTAGGAAGCTC | 20901 | 0.1699823015413323 | No Hit |
| ACTTGATCCCAACTCATCTCTCATTTATTTCGGCTTCTTTTATTCCAGGA | 20655 | 0.1679816486453384 | No Hit |
| TGGCTCTCCCAGAGGCAGAAGGACAATGGCTGTTTCAGGAGCTCTGGGTC | 20569 | 0.1672822334052755 | No Hit |
| TGTTGTGCTAAACGCTGGGAGGGTACAAGTTGGTCATTCCTAAATCTGTG | 20212 | 0.16437884688547955 | No Hit |
| TTTACACCTGCTAGGGTGTTCAAAGGTCAGTGCTATAGAAATTCAGTATC | 19748 | 0.1606052576832797 | No Hit |
| TCCCTTTCTCCATGCATGAATTTTCAATGCAGACAGCTGTACGATATACG | 18757 | 0.1525457169518573 | No Hit |
| CTAAGTGCTGTGTTGTCGTTCCCCCTGCTTAAAATAAAGTTGTTTCTTAA | 18610 | 0.15135020485547074 | No Hit |
| TGTCCTATAACAAGCTTAAAAACATACCAACTGTCAATGAAAACCTTGAA | 17211 | 0.1399725081014243 | No Hit |
| AACATCTGTGTTGGGGAGAGTGGAGACAGACTGACGCGAGCAGCCAAGGT | 16931 | 0.13769534220354512 | No Hit |
| ATTCTTCCTACCCATGAGCATGGAATGTTCTTCCATTTGTTTGTATCCTC | 16610 | 0.13508473415633362 | No Hit |
| CGGTTGTACTTGCGGATGTAGTGCAGATAGTCTCGGCGGATGACAATGGT | 16563 | 0.13470249559490388 | No Hit |
| TTCCCCCTGCGCATGCGGGACTGGCTCAAGAACGTCCTGGTCACCCTGTA | 16290 | 0.13248225884447168 | No Hit |
| TGCATATACTGGTTAGTGAGGTGAGCCTGGCGCTCTTCTTTGCGCTGAGC | 16156 | 0.1313924723076295 | No Hit |
| ATTTTCCCAGGTGGGGCCTGCCTGGGGAAAGCTTGTGGCCGGAAGAGAAA | 15853 | 0.12892825349671022 | No Hit |
| GGGTACTTCAGGGTCAGGATGCCACGCTTGCTCTGGGCCTCGTCGCCCAC | 15394 | 0.12519532797125826 | No Hit |
| TGCGAAAAGTTACTTTCAATGGACTGAACCAGATGATTGTCATAGAACTG | 14884 | 0.1210476329429783 | No Hit |
| TGTCCATGTCTTACTACTTTGACCGCGATGATGTGGCTTTGAAGAACTTT | 14346 | 0.11667222132491041 | No Hit |
| ATTCGAGGGCGGATCCTCTCTGGCGTGGTGACCAAGATGAAGATGCAGAG | 13942 | 0.11338659624368472 | No Hit |
| ACAGTGTATCTAGCTTTGGAAAACACAGGGGTCTGCCCTGTGAGCTGCTC | 13443 | 0.10932836130425003 | No Hit |

## Adapter Content

Produced by FastQC (version 0.11.7)
